# Life Course Socioeconomic Position: Associations with Cardiac Structure and Function at Age 60-64 Years in the 1946 British Birth Cohort

**DOI:** 10.1371/journal.pone.0152691

**Published:** 2016-03-31

**Authors:** Emily T. Murray, Rebecca Jones, Claudia Thomas, Arjun K. Ghosh, Naveed Sattar, John Deanfield, Rebecca Hardy, Diana Kuh, Alun D. Hughes, Peter Whincup

**Affiliations:** 1 Population Health Research Centre, Division of Population Health Sciences and Education, St George's University of London, London, United Kingdom; 2 National Heart and Lung Institute, Imperial College Academic Health Sciences Centre, London, United Kingdom; 3 British Heart Foundation Glasgow Cardiovascular Research Centre, University of Glasgow, Glasgow, United Kingdom; 4 Vascular Physiology Unit, Institute of Cardiovascular Science, University College London, London, United Kingdom; 5 MRC Unit for Lifelong Health and Ageing, at University College London, London, United Kingdom; 6 Institute of Cardiovascular Science, University College London, London, United Kingdom; Providence VA Medical Center and Brown University, UNITED STATES

## Abstract

Although it is recognized that risks of cardiovascular diseases associated with heart failure develop over the life course, no studies have reported whether life course socioeconomic inequalities exist for heart failure risk. The Medical Research Council’s National Survey of Health and Development was used to investigate associations between occupational socioeconomic position during childhood, early adulthood and middle age and measures of cardiac structure [left ventricular (LV) mass index and relative wall thickness (RWT)] and function [systolic: ejection fraction (EF) and midwall fractional shortening (mFS); diastolic: left atrial (LA) volume, E/A ratio and E/e’ ratio)]. Different life course models were compared with a saturated model to ascertain the nature of the relationship between socioeconomic position across the life course and each cardiac marker. Findings showed that models where socioeconomic position accumulated over multiple time points in life provided the best fit for 3 of the 7 cardiac markers: childhood and early adulthood periods for the E/A ratio and E/e’ ratio, and all three life periods for LV mass index. These associations were attenuated by adjustment for adiposity, but were little affected by adjustment for other established or novel cardio-metabolic risk factors. There was no evidence of a relationship between socioeconomic position at any time point and RWT, EF, mFS or LA volume index. In conclusion, socioeconomic position across multiple points of the lifecourse, particularly earlier in life, is an important determinant of some measures of LV structure and function. BMI may be an important mediator of these associations.

## Introduction

Chronic heart failure is a common and important manifestation of cardiovascular disease in older people, with a poor prognosis [1–2]. Heart failure with preserved ejection fraction is an increasingly recognised problem [3–4], with changes in left ventricular (LV) structure and function providing early indicators of future likelihood of heart failure [1] and mortality [5–6].

Recent studies have emphasized that socioeconomic position in adulthood is related to the development of heart failure, heart failure risk being 30–50% higher in lower socioeconomic groups [7]. Although risks of specific cardiovascular diseases associated with heart failure, particularly coronary heart disease (CHD), develop over the life course [8], and are influenced by socioeconomic position from childhood onwards [9–11], only one study has so far reported on the influence of socioeconomic position at different stages of the life course on the risk of heart failure [12]. Markers of left ventricular mass and left ventricular function, both systolic and diastolic, are important predictors of heart failure risk [1]; recent reports have suggested that markers of low SEP, particularly low educational attainment, is associated with higher LV mass [13–14], and with impaired LV systolic and diastolic function [13]. However, there are no reported studies linking life course socioeconomic position to LV structure and function. Defining the associations between socioeconomic position at different stages of the life course and predictors of heart failure could help to investigate the biological processes responsible for the social patterning of cardiac failure. This would require the heart to be viewed in terms of its structure and geometry (reflecting the loads that it has experienced), as well as systolic and diastolic function. The ability to address this type of question therefore necessitates a range of cardiac measures and also requires information on the potential behavioural and biological mediators of these relationships, to help link the pathways by which social influences impact on the biological. We have therefore investigated the relationship between socioeconomic position at three separate points during the life course (childhood, early adulthood and middle age) and measures of LV structure and dysfunction (both systolic and diastolic) at 60–64 years, using a structured modelling approach to assess whether relationships are best explained by sensitive period, accumulation or social mobility models [15]. We have also examined whether life course associations were mediated by risk factors implicated in the development of heart failure, including adiposity, blood pressure, heart rate, alcohol intake or cardio-metabolic risk factors implicated in the development of heart failure, including adipokines, inflammatory markers and proinsulin [16–18].

## Materials and Methods

### Study design

The MRC National Survey of Health and Development is a socially stratified British cohort of 5,362 men and women followed up at regular intervals between birth during one week in March 1946 and late middle age [19]. Between 2006 and 2010 (aged 60-64), 2,856 study members (those still alive and with a known address in England, Scotland or Wales) were invited for assessment at a clinical research facility or a research nurse home visit. Invitations were not sent to participants who had died (778), were living abroad (570), had previously withdrawn from the study (594) or had been lost to follow-up (564). Of those invited, 2,229 (78%) were assessed: 1690 at a clinic and 539 at home [20].

### Cardiac measures

Trained and accredited echocardiographers made cardiac measurements from participants attending a clinic, in accordance with American Society of Echocardiography guidelines using a GE Vivid I ultrasound scanner [21]. Images from parasternal long axis and short axis views, apical 4-chamber, 5-chamber, 3-chamber, 2-chamber and aortic views (including tissue Doppler studies in the 4-chamber view) were recorded. A range of parameters representing LV structure [(LV mass and relative wall thickness (RWT)], LV systolic dysfunction [ejection fraction—Teichholz method (EF) and midwall fractional shortening (mFS)] and LV diastolic disfunction [left atrial (LA) volume, E/e’ ratio (non-invasive estimate of LV filling pressure) and E/A ratio (early to late diastolic mitral inflow velocities) were calculated. Both LV mass and LA volume were indexed to height^2.7^ for analysis, as this indexation performs better in the context of overweight/obesity [22].

### Socioeconomic position

Measures of socioeconomic position, defined by the Registrar General’s six-level occupational classification scheme [23], were selected for analysis at three points over the life course. The occupation of the father, or step-father, when the study member was aged 4 was chosen to represent childhood socioeconomic position, while head of household occupation at ages 26 and 53 represented socioeconomic position in early adulthood and middle age. Missing values were imputed from adjacent ages (33 values from age 7 and 14 values from age 11 for childhood socioeconomic position; 107 values from age 36 for early adulthood; 107 values from age 43 for late adulthood).

### Covariates

All covariates were assessed at 60–64 years. Body mass index was calculated from measured height and weight (kg/m^2^). Seated blood pressure was measured using an Omron HEM-705 oscillometric recorder. Self-reported alcohol intake for the previous week was classified into five categories: abstainer, very light, light, moderate or heavy drinker (0, <1, <2, <4 or 4+ drinks per day respectively). Overnight fasting blood samples were taken during the clinic or home visit and initially processed at the clinic laboratories. Aliquots were frozen and stored before transfer to central laboratories (MRC Human Nutrition Research laboratory, Cambridge and British Heart Foundation Research Centre, Glasgow) for measurement of low-density and high-density lipoprotein cholesterol (LDL and HDL), glycated haemoglobin (HbA1c), C-reactive protein, Interleukin-6, E-selectin, tissue-plasminogen activator, proinsulin, leptin and adiponectin. Assay methods and interassay coefficients of variation are given in S1 Table.

### Statistical Analysis

Associations between socioeconomic position and each cardiac measure were initially investigated using separate linear regression models for socioeconomic position at each time point, Bonferroni-adjusted for the multiple testing of 7 outcomes. Linearity of associations was assessed using scatterplots and Lowess smoothed curves using 6-category SEP categories. Due to small numbers in categories I and VI, measures were re-categorized into four levels for presentation: professional and intermediate (I and II); skilled non-manual (IIInm); skilled manual (IIIm); and other manual (V and VI). Analyses also indicated that patterns were not different for men and women, so analyses were performed on data from all participants adjusting for sex.

Next, a structured modelling approach [15] was used to examine different hypothesized life course socioeconomic position models in relation to each cardiac measure. The basic premise of the approach is to compare the model fit of a set of nested life course models to a saturated model containing all possible main effects and interactions. A p-value >0.05 (statistically significant) indicated that there was no evidence that the more complex model explained the data better than the simpler life course model, and the latter model should be adopted. For each cardiac measure, the life course model with the highest p-value was chosen as the best fitting model for that measure. The life course models considered were as follows: (1) sensitive periods—childhood, early adulthood or middle age; (2) accumulation—low socioeconomic position in early life only, in adulthood only or across all three time points; and (3) social mobility—upward or downward mobility in adulthood only or between any of the three time points (model specifications–S2 Table). To avoid small cell counts, socioeconomic position at each time period was further collapsed into binary indicators representing manual or non-manual occupation.

Subsequently, we fitted the identified life course socioeconomic position model to obtain estimates of mean differences in the specified cardiac measure for the relevant exposure to manual socioeconomic position(s). These models were then adjusted for several cardiovascular risk factors, fitted as continuous variables; first separately and then simultaneously.

Sensitivity analysis was conducted for all stages of analyses replacing LV mass and LA volume indexed to height^2.7^ with an index of height^1.7^, an index of body surface area and with no index. To investigate possible bias due to missing data, the models were also refitted using multiple imputation. Fifty imputed datasets were obtained via chained equations using 50 cycles per dataset.^23^ All analyses were performed using Stata 12 (StataCorp 2011).

## Results

Of 2,229 participants studied at 60–64 years, 73.5% had data available for one or more cardiac risk markers. Of these, all had a measure of socioeconomic position for at least one age and were therefore included in the initial analyses. Participant characteristics are presented in Table 1. Included participants (n = 1638), compared to excluded (n = 591)(mainly lack of echocardiography at home visit), were more likely to be in a non-manual socioeconomic position in childhood (50.0% vs. 33.3%) young adulthood (71.6% vs. 52.5%) and later adulthood (76.7% vs. 53.8%), less likely to be a current smoker (9.2% vs. 13.3%) or alcohol abstainer (19.6% vs. 33.8%) (data not shown).

**Table 1:** Description of the study population.

Variables	Total n	Mean (standard deviation)			p-value for gender difference
		All (n = 1638)	Men (n = 790)	Women (n = 848)	
*Cardiac measures*:					
LV mass index (g/m^2.7^)	1479	44.2 (13.3)	46.1 (13.8)	42.4 (12.5)	*<0*.*001*
Relative wall thickness (RWT)	1479	0.42 (0.09)	0.42 (0.09)	0.41 (0.09)	*0*.*06*
LV ejection fraction (EF) (%)	1493	68.5 (10.4)	67.0 (10.8)	69.8 (9.7)	*<0*.*001*
Midwall fractional shortening (mFS) (%)	1475	17.1 (3.3)	16.7 (3.3)	17.5 (3.3)	*<0*.*001*
Left atrial (LA) volume index (ml/ m^2.7^)	1408	9.7 (3.5)	9.7 (3.5)	9.7 (3.6)	*0*.*814*
E/A ratio	1577	1.00 (0.28)	1.00 (0.28)	0.99 (0.28)	*0*.*50*
E/e´	1491	7.9 (2.1)	7.5 (2.0)	8.3 (2.1)	*<0*.*001*
Ejection fraction EF < 40% n, (%)	1493	25 (1.7)	16 (2.3)	9 (1.1)	*0*.*095*
*Social Class measures*:					
*Childhood*, *n (%)*					
I and II	1554	439 (26.8)	216 (27.3)	223 (26.3)	
IIInm		338 (20.6)	160 (20.3)	178 (21.0)	
IIIm		428 (26.1)	208 (26.3)	220 (25.9)	
IV and V		349 (21.3)	170 (21.5)	179 (21.1)	*0*.*861*
*Early adult*, *n (%)*					
I and II	1548	695 (44.9)	342 (45.7)	353 (44.2)	
IIInm		329 (21.3)	130 (17.4)	199 (24.9)	
IIIm		369 (23.8)	201 (26.8)	168 (21.0)	
IV and V		155 (10.0)	76 (10.2)	79 (9.9)	*0*.*001*
*Late adult*, *n (%)*					
I and II	1615	955 (59.1)	484 (62.4)	471 (56.1)	
IIInm		198 (12.3)	80 (10.3)	118 (14.1)	
IIIm		313 (19.4)	159 (20.5)	154 (18.4)	
IV and V		149 (9.2)	53 (6.8)	96 (11.4)	*0*.*001*
*Established risk factors*:					
Fasting LDL-cholesterol (mmol/L)	1528	3.5 (1.0)	3.3 (0.9)	3.7 (1.0)	*<0*.*001*
Fasting HDL-cholesterol (mmol/L)	1536	1.6 (0.4)	1.4 (0.3)	1.8 (0.4)	*<0*.*001*
Systolic blood pressure (mmHg)	1636	135.7 (18.0)	139.0 (17.8)	132.6 (17.6)	*<0*.*001*
Diastolic blood pressure (mmHg)	1636	77.3 (9.7)	79.0 (9.8)	75.7 (9.3)	*<0*.*001*
Body mass index (kg/m^2^)	1640	27.6 (4.6)	27.7 (4.0)	27.6 (5.2)	*0*.*42*
Glycated haemoglobin	1535	5.8 (0.6)	5.8 (0.7)	5.8 (0.6)	*0*.*86*
*Diabetes*, *n (%)*	1362	130 (9.5)	79 (12.1)	51 (7.2)	*0*.*002*
*Smoking*, *n (%)*					
Current	1504	145 (9.6)	75 (10.4)	70 (9.0)	
Ex-smoker		621 (41.3)	350 (48.4)	271 (34.7)	
Never smoked		738 (49.1)	298 (41.2)	440 (56.3)	*<0*.*001*
*Alcohol consumption*, *n (%)*					
Abstainers (0 drinks per day)	1622	318 (19.6)	106 (13.5)	212 (25.3)	
Very light drinkers (<1)		648 (40.0)	223 (28.5)	425 (50.7)	
Light drinkers (1-<2)		324 (20.0)	189 (24.1)	135 (16.1)	
Moderate drinkers (2-<4)		240 (14.8)	179 (22.9)	61 (7.3)	
Heavy drinkers (4+)		92 (5.7)	86 (11.0)	6 (0.7)	*<0*.*001*
*Novel risk factors*:					
C-reactive protein	1572	3.8 (8.7)	3.8 (9.3)	3.7 (8.0)	*0*.*86*
Interleukin-6	1569	2.6 (2.6)	2.7 (2.7)	2.5 (2.5)	*0*.*09*
Leptin (ng/ml)	1570	18.3 (20.8)	9.6 (8.5)	26.6 (25.1)	*<0*.*001*
Adiponectin (*μg*/ml)	1570	14.9 (10.2)	10.3 (7.0)	19.2 (10.9)	*<0*.*001*
E-selectin (ng/ml)	1568	39.1 (18.3)	40.7 (19.2)	37.6 (17.3)	*<0*.*001*
Tissue plasminogen activator (ng/ml)	1527	10.0 (5.4)	10.6 (5.6)	9.4 (5.1)	*<0*.*001*
Fasting Proinsulin (pmol/L)	1527	11.1 (11.3)	13.0 (13.5)	9.3 (8.2)	*<0*.*001*

I and II, professional and intermediate; IIInm, skilled non-manual; IIIm, skilled manual; IV and V, other manual

Individuals in lower socioeconomic position groups had more adverse levels of LV mass index (higher), RWT (lower), E/A ratio (lower) and E/e’ ratio (higher), but the points in the life course at which SEP was associated with each cardiac measures differed (Table 2). LV mass index was associated with socioeconomic position at all points across the life course, with the strength of these associations similar at each time point. In contrast, E/e’ ratio was associated with socioeconomic position in childhood and middle age (but not in early adulthood), while E/A ratio was associated with socioeconomic position only in childhood (but midlife SEP p-value = 0.05). There was no evidence of associations between socioeconomic position at any of the three time points and EF, mFS or LA volume.

**Table 2:** Sex- and age-adjusted differences (95% CI) in cardiac markers by socioeconomic position at three points during the lifecourse.

	LV Structure		Systolic Function		Diastolic Function		
	LV Mass Index (g/m ^2.7^)	RWT	EF	mFS (%)	LA volume index (ml/m^2.7^)	E/A ratio	E/e’ ratio
	(N = 1,477)	(N = 1,477)	(N = 1,491)	(N = 1,473)	(N = 1,406)	(N = 1,575)	(N = 1,489)
**Childhood**							
I and II	–	–	–	–	–	–	–
IIInm	0.33 (1.60, 2.26)	0.00 (-0.02, 0.01)	-0.28 (-1.79, 1.23)	0.01 (-0.48, 0.50)	1.14 (-1.05, 3.33)	0.00 (-0.04, 0.04)	0.07 (-0.23, 0.38)
IIIm	3.79 (1.97, 5.61)	0.00 (-0.01, 0.01)	-0.75 (-2.18, 0.68)	-0.28 (-0.75, 0.18)	1.40 (-0.65, 3.44)	-0.06 (-0.10, -0.02)	0.56 (0.27, 0.85)
IV and V	4.30 (2.37, 6.22)	0.00 (-0.01, 0.01)	0.06 (-1.45, 1.57)	-0.13 (-0.62, 0.36)	2.07 (-0.10, 4.24)	-0.06 (-0.10, -0.02)	0.39 (0.08, 0.70)
*Trend p* ^a^	*<0*.*0001*	*0*.*99*	*0*.*99*	*0*.*99*	*0*.*18*	*0*.*001*	*0*.*002*
**Early Adulthood**							
I and II	–	–	–	–	–	–	–
IIInm	0.96 (-0.82, 2.74)	0.00 (-0.01, 0.01)	0.64 (-0.74, 2.03)	0.22 (-0.23, 0.67)	0.58 (-1.47, 2.62)	0.00 (-0.03, 0.04)	0.37 (0.08, 0.65)
IIIm	4.58 (2.86, 6.31)	-0.01 (-0.02, 0.00)	0.26 (-1.08, 1.61)	0.33 (-0.10, 0.77)	0.89 (-1.07, 2.86)	-0.03 (-0.07, 0.00)	0.59 (0.32, 0.87)
IV and V	4.76 (2.34, 7.17)	-0.01 (-0.02, 0.01)	0.62 (-1.24, 2.48)	0.12 (-0.48, 0.73)	3.54 (0.81, 6.27)	-0.03 (-0.08, 0.02)	0.37 (-0.01, 0.74)
*Trend p* ^a^	*0*.*001*	*0*.*33*	*0*.*99*	*0*.*73*	*0*.*08*	*0*.*17*	*<0*.*001*
**Middle Age**							
I and II	–	–	–	–	–	–	–
IIInm	0.91 (-1.19, 3.00)	-0.01 (-0.02, 0.01)	-0.19 (-1.82, 1.43)	-0.19 (-0.34, 0.72)	-1.33 (-3.70, 1.05)	0.00 (-0.05, 0.04)	0.28 (-0.06, 0.62)
IIIm	3.51 (1.76, 5.26)	-0.02 (-0.03, 0.00)	-0.24 (-1.60, 1.12)	0.39 (-0.05, 0.84)	1.57 (-0.39, 3.53)	-0.03 (-0.06, 0.01)	0.22 (-0.06, 0.49)
IV and V	4.78 (2.45, 7.11)	0.01 (-0.01, 0.02)	-0.38 (-1.43, 2.20)	-0.07 (-0.66, 0.52)	0.47 (-3.31, 2.17)	-0.06 (-0.10, -0.01)	0.27 (-0.10, 0.64)
*Trend p* ^a^	*<0*.*0001*	*0*.*99*	*0*.*99*	*0*.*99*	*0*.*99*	*0*.*05*	*0*.*14*

I and II, professional and intermediate; IIInm, skilled non-manual; IIIm, skilled manual; IV and V, other manual; E/A, early to late ventricle fill; E/e’, early filling to early diastolic mitral annular velocity; EF, ejection fraction; LA, left atrial; LV, left ventricular; mFS, midwall fractional shortening; RWT, relative wall thickness.

^a^ Bonferroni adjusted p-value to adjust for multiple tests.

Of the original sample used in this analysis, 1,456 (88.9%) had measures of socioeconomic position at all three times, permitting socioeconomic position trajectories from birth to middle age to be ascertained (S3 Table). Table 3 displays the formal examination of each life course model’s ability to describe the relationships between socioeconomic position and each cardiac measure. In these models, only LV mass index, E/e’ ratio and E/A ratio had associations with life course socioeconomic position. For both LV mass index, the whole life accumulation model offered the closest fit to the saturated model, ahead of the early life accumulation model. For E/A ratio and E/e’ ratio, the early life accumulation model provided a slightly better fit of the saturated model over the childhood sensitive period or whole life accumulation model. There was no evidence for an interaction between socioeconomic position and sex in any of the selected models.

**Table 3:** Sex and age-adjusted p-values from partial F tests comparing each life course model with the saturated model for the associations between socioeconomic position and selected cardiac markers ^a^.

	LV Structure		Systolic Function		Diastolic Function		
Life course model	LV Mass Index (g/m ^2.7^)	RWT	EF	mFS (%)	LA Volume Index (ml/m^2.7)^	E/A ratio	E/e’ ratio
No effect	<0.001	0.728*	0.598*	0.371*	0728*	0.001	<0.001
**Sensitive period models**							
Childhood	**0.001**	0.619	**0.647**	**0.459**	**0.778**	0.169*	0.031
Early adulthood	0.016	**0.978**	**0.500**	**0.403**	**0.886**	0.005	0.014
Middle age	0.001	**0.700**	**0.500**	**0.283**	**0.945**	0.003	<0.001
**Accumulation model**							
Childhood and early adulthood	**0.352**	**0.760**	**0.509**	**0.273**	**0.919**	**0.181**	**0.278**
Early adulthood and middle age	**0.150**	**0.906**	**0.481**	**0.347**	**0.977**	0.009	0.005
Whole life	0.980*	**0.777**	**0.514**	**0.271**	**0.984**	**0.144**	0.093*
**Social mobility models**							
Adulthood	<0.001	**0.715**	**0.473**	**0.364**	**0.491**	<0.001	<0.001
Whole life	<0.001	**0.633**	**0.434**	**0.376**	**0.514**	0.001	<0.001

Abbreviations: E/A, early to late ventricle fill; E/e’, early filling to early diastolic mitral annular velocity; EF, ejection fraction; LA, left atrial; LV, left ventricular; mFS, midwall fractional shortening; RWT, relative wall thickness.

^a^ Larger p values represent better model fit. Bolded text indicates a p-value >0.05. Asterisk indicates the selected model–the most parsimonious model with a good fit to the data.

Modelling the selected life course model for cardiac measures, except when the no effect model was chosen (Table 4), showed that after adjustment for age and sex, LV mass index increased by 2.48 g/m2.7 (95% CI: 1.75 to 3.22) for each time spent in a manual socioeconomic position (out of 3 possible). E/A ratio was -0.040 (95% CI: -0.061 to -0.019) lower and E/e’ ratio 0.36 (0.21, 0.52) higher for each time spent in manual occupation during childhood and early adulthood. Further adjustment for current BMI attenuated associations by 32%, 28% and 19%, respectively. Adjustment for other established risk factors of blood pressure, alcohol consumption, smoking, cholesterol, heart rate or hbA1c had only small effects on estimates. Additional adjustment for E-selectin, t-PA, proinsulin, leptin and adiponectin did not substantially affect associations.

**Table 4:** Mean difference (95% CI) in each cardiac measure for selected life course socioeconomic position model, individually adjusted for potential mediators (not cumulatively).

	Whole life accumulation model^a^	Childhood and early adulthood accumulation model^b^	
	LV Mass Index (g/m ^2.7^)	E/e’ ratio	E/A ratio
	N = 1,034	(N = 1,093)	(N = 1,098)
Age and sex adjusted	2.48 (1.75, 3.22)	0.36 (0.21, 0.52)	-0.040 (-0.061, -0.019)
+ Body mass index	1.69 (1.01, 2.37)	0.29 (0.13, 045)	-0.029 (-0.050, -0.008)
+ Blood pressure (systolic & diastolic)	2.36 (1.63, 3.08)	0.33 (0.18, 0.49)	-0.040 (-0.060, -0.019)
+ Alcohol	2.44 (1.70, 3.18)	0.35 (0.20, 0.51)	-0.041 (-0.062, -0.200)
+ Smoking	2.37 (1.62, 3.12)	0.36 (0.20, 0.52)	-0.040 (-0.061, -0.019)
+ Cholesterol (LDL and HDL)	2.20 (1.47, 2.94)	0.33 (0.18, 0.49)	-0.036 (-0.057, -0.014)
+ Heart Rate	2.47 (1.73, 3.20)	0.35 (0.20, 0.51)	-0.039 (-0.060, -0.018)
+ Glycated Hemoglobin (hbA1c)	2.41 (1.67, 3.14)	0.37 (0.21, 0.52)	-0.038 (-0.058, -0.018)
+ All Established Risk Factors	1.46 (0.78, 2.14)	0.25 (0.09, 0.41)	-0.030 (-0.050, -0.010)
+ CRP	2.48 (1.74, 3.22)	0.37 (0.21, 0.52)	-0.040 (-0.061, -0.019)
+ IL-6	2.47 (1.74, 3.21)	0.36 (0.21, 0.52)	-0.039 (-0.060, -0.018)
+ E-selectin	2.40 (1.66, 3.13)	0.35 (0.19, 0.51)	-0.039 (-0.060, -0.018)
+ t-PA	2.47 (1.73, 3.20)	0.36 (0.20, 0.51)	-0.037 (-0.058, -0.017)
+ Proinsulin	2.33 (1.59, 3.07)	0.33 (0.17, 0.48)	-0.038 (-0.059, -0.017)
+ Leptin	2.34 (1.61, 3.07)	0.35 (0.20, 0.51)	-0.037 (-0.058, -0.016)
+ Adiponectin	2.36 (1.63, 3.10)	0.36 (0.20, 0.52)	-0.036 (-0.057, -0.015)
+ All Novel Cardio-metabolic Risk Factors	2.17 (1.44, 2.90)	0.32 (0.17, 0.48)	-0.032 (-0.053, -0.011)
+ All Risk Factors	1.47 (079, 2.15)	0.24 (0.09, 0.40)	-0.030 (-0.050, -0.010)

^a^ Each additional time point (0–3) in manual vs non-manual social class

^b^ Each additional time point (0–2) in manual vs non-manual social class during childhood and young adulthood.

CRP, c-reactive protein; E/A, early to late ventricle fill; E/e’, early filling to early diastolic mitral annular velocity; HDL, high-density lipoprotein; IL-6, interleukin-6; LDL, low-density lipoprotein; LV, left ventricular; t-PA, tissue plasminogen activator.

Sensitivity analyses using alternative indexes, and no index, for LV mass and LA volume, did not qualitatively change results (data not shown). Results using multiple imputation (S4 Table) were similar to those of the complete case analysis, with the exception that for E/e’ ratio the p-value for the childhood and early adulthood life course model was slightly higher than the p-value for the whole life accumulation model and that estimates for relationships were slightly lower, but still qualitatively unchanged, compared to the complete case analysis.

## Discussion

In this prospective birth cohort study, using a novel analytic approach, we showed that socioeconomic position across multiple periods in the life course was associated with some measures of LV structure and diastolic function, independent of established and newer cardio-metabolic risk markers measured at 60–64 years. Associations were attenuated considerably by adjustment for BMI, less so by other CHD risk factors. There was no evidence that socioeconomic position was related to relative wall thickness, ejection fraction, midwall fractional shortening or LA volume index.

To our knowledge, this is the first study to show that differences in occupational social class at different stages in the life course are related to measures of cardiac structure and function. Two previous studies [13–14] found that lower educational achievement was related to higher LV mass index, while in only the latter study [13] education was also related to a higher prevalence of low LV ejection fraction and severe diastolic dysfunction. Although several studies have examined the associations of adult socioeconomic position with heart failure development, hospitalisation and mortality [7], none has examined particular associations with LV systolic or diastolic dysfunction [24].

The finding that socioeconomic position at different points in the life course was associated with LV mass index, E/A ratio and E/e’ but not with RWT, EF, mFS or LA volume is novel, and points to socioeconomic effects predominantly being associated with increased load on the left ventricle and with diastolic, rather than systolic, ventricular dysfunction. The lack of a discernible association between socioeconomic influences and LA volume index could reflect either the limited duration and/or extent of diastolic dysfunction in a population-based sample [25] or the imprecision in the measurement of LA volume by echocardiography [26]. The strong associations with LV mass may be particularly important, as this has been shown to predict cardiovascular events independent of other risk factors, including blood pressure [27].

One recent study [12] did find an association between adult, but not childhood, socioeconomic position and incident heart failure. Although their measure of heart failure was not separated into systolic or diastolic failure, their finding that a composite measure of social class using occupation, education, housing tenure, pension and amenities had a stronger association with incident heart failure than occupational class alone, could suggest that the strength of association between adult SEP and cardiac markers are underestimated; with implications that the best fitting life course models for E/A ratio and E/e’ could easily match that of LV mass index of the whole life accumulation model.

In further analysis, BMI had the largest impact on the associations between socioeconomic position and LV mass index, E/A ratio and E/e’ ratio, reducing their strengths appreciably. Patients with LV diastolic dysfunction and heart failure with preserved ejection fraction (‘diastolic heart failure’) do also tend to have a chronic pro-inflammatory state induced by disease co-morbidities, particularly obesity [28], which is associated with low SEP which occurs more frequently in lower social classes [29–31]. However, further adjustment for inflammatory markers did not substantially alter these relationships. This suggests that obesity may represent an important pathway linking SEP with LV diastolic dysfunction and is consistent with other evidence of associations between obesity and heart failure [18]. However, the findings also suggest that inflammatory and haemostatics markers are not representing an important biological pathway, even in the presence of obesity. In addition, there was no additional reduction in the strengths of SEP-cardiac associations when adjusting for other cardio-metabolic risk markers (including several established risk markers for cardiac failure), suggesting that these markers are not on the pathway from socio-economic position to cardiac structure and function. Other potential mechanisms which could link SEP with cardiac structure and function include direct effects of social hierarchy [32], other unhealthy behaviours in lower socioeconomic groups [33] or increased stress from lower job control found in lower social class occupations [34]; all of which are related to cardiovascular disease risk [35–36].

The strengths of this study include availability of detailed data on cardiac structure and function in a large birth cohort in late middle aged and with prospectively collected data on socioeconomic position over the life course. The cohort is still relatively healthy, suggesting that if relationships shown here are true, that associations may strengthen as the cohort ages. For E/A in particular, LV filling patterns show a U-shaped relation with LV diastolic function—declining in early diastolic dysfunction, undergoing ‘pseudo-normalization’ with moderate disease and can be elevated in severe ‘restrictive’ disease [37]–meaning that relationships between socioeconomic position and E/A may be particularly vulnerable to underestimation if there is substantial ‘pseudo-normalization’.

The structured modelling approach we used to compare several different life course socioeconomic position models is an improvement over traditional regression models where results are interpreted from a single pre-specified model without considering the merits of alternative models [16]. However, the method is limited by the need to dichotomize socioeconomic position categories, which could potentially result in relationships being obscured if there was not a consistent trend across social class categories. It would be possible to increase the number of socioeconomic position categories in the life course models, but this would result in loss of statistical power, especially where interactions are involved (i.e. social mobility models). Inclusion of only participants with complete cardiac and socioeconomic data reduced sample size but bias appeared unlikely as excluded participants did not differ markedly from included, and results of imputation analyses were very similar to those of complete case analysis. As in any longitudinal study, attrition of the sample occurred, despite high response rates [19]. Selection bias is likely to be present in our sample, as cohort members with lower socioeconomic positions in childhood and young adulthood ages were more likely not to have data on one of the studied cardiac outcomes the higher social class peers (data available from authors). This suggests that study members with early heart failure, who were more likely to be of lower socioeconomic position, were under-represented in the analysis and our findings under-estimates of true causal associations. In conclusion, socioeconomic disadvantage across life, particularly earlier in life, has persistent long-term adverse effects on some measures of cardiac structure and function, particularly markers of left ventricular mass and diastolic dysfunction. Adult adiposity appears to play an important role as a mediator of the associations between childhood social class and the cardiac measures but does not fully explain them. Prevention of obesity in low socioeconomic groups starting in childhood could play an important role in reducing inequalities in heart failure in adult life.

## Supporting Information

S1 Table Methods and inter-assay coefficients of variation (CV) for cardio-metabolic risk factors assessed from blood samples. (DOCX)

S2 Table Model specification and constraints for given life course models. (DOCX)

S3 Table Means (standard deviations) of cardiac measures by social class trajectory over the life course. (DOCX)

S4 Table Sex- and age-adjusted differences (95% CI) in cardiac markers by socioeconomic position at three points during the life course, after multiple imputation. (DOCX)
