# Supplementary material for: Life Course Socioeconomic Position: Associations with Cardiac Structure and Function at Age 60-64 Years in the 1946 British Birth Cohort
Source: PLoS One. 2016 Mar 31;11(3):e0152691. doi: 10.1371/journal.pone.0152691 (PMC4816291; doi:10.1371/journal.pone.0152691)
Supplement: S1 Table — (DOCX) [file pone.0152691.s001.docx]

**S1 Table:** Methods and inter-assay coefficients of variation (CV) for cardio-metabolic risk factors assessed from blood samples.

| **Risk factor** | **Units** | **Assay/Method** | **CV (%)** |
| --- | --- | --- | --- |
| Low-density lipoprotein cholesterol (LDL) | Mmol/L | Total cholesterol – HDL cholesterol – (triglycerides/2.2) | n/a |
| High-density lipoprotein cholesterol (HDL) | Mmol/L | Cholesterol oxidase, couple with peroxidase | 1.7% at 1.14 nmol/L |
| Glycated haemoglobin (hbA1c) | % | TOSOH G7 analyser | 1.42% at c5.67 |
| C-reactive protein (CRP) | mg/l | Particle-enhanced immunoturbidimetric assay | 4.3% at 3.4 mg/L  1.8% at 11.9 mg/L |
| Interleukin-6 (IL6) | pg/ml | Enzyme-linked immunosorbent assay (ELISA) | 6.5% |
| E-selectin | ng/ml | High sensitivity ELISA | <10.0% |
| Tissue plasminogen activator (tPA) | ng/ml | Enzyme-linked immunosorbent assay (ELISA) | 6.6% |
| Proinsulin | Pmol/L | Enzyme-linked immunosorbent assay (ELISA) | <5.0% |
| Leptin | ng/ml | In-house radioimmunoassay validated against commercially available assays | <10.0% |
| Adiponectin | ug/ml | Enzyme-linked immunosorbent assay (ELISA) | <7.5% |
