# Supplementary material for: Life Course Socioeconomic Position: Associations with Cardiac Structure and Function at Age 60-64 Years in the 1946 British Birth Cohort
Source: PLoS One. 2016 Mar 31;11(3):e0152691. doi: 10.1371/journal.pone.0152691 (PMC4816291; doi:10.1371/journal.pone.0152691)
Supplement: S2 Table — (DOCX) [file pone.0152691.s002.docx]

**S2 Table:** Model specification and constraints for given life course models.

|  | Life-course model specification | Constraints |
| --- | --- | --- |
| Saturated model | α + b_1_S_1_ + b_2_S_2_ + b_3_S_3_ + θ_12_S_1_S_2_+ θ_13_ S_1_S_3_ + θ_23_S_2_S_3_+ θ_123_S_1_S_2_S_3_ |  |
| No effect | α |  |
| **Sensitive period models** |  |  |
| Childhood (age 4) | α + b_1_S_1_ |  |
| Early adulthood (age 26) | α + b_2_S_2_ |  |
| Middle age (age 53) | α + b_3_S_3_ |  |
| **Accumulation models** |  |  |
| Childhood and early adulthood | α + S_1_ (b_1_ + b_2_) |  |
| Early adulthood and middle age | α + S_1_ (b_2_ + b_3_) |  |
| Whole life | α + S_1_ (b_1_ + b_2_ + b_3_) |  |
| **Social mobility models** |  |  |
| Adulthood (age 26 to 53) | α + b_2_S_2_ + b_3_S_3_ + θ_23_S_2_S_3_ | θ_23_ S_2_S_3_ = - (b_2_ + b_3_) |
| Whole life | α + b_1_S_1_ + b_2_S_2_ + b_3_S_3_ + θ_12_S_1_S_2_+ θ_23_ S_2_S_3_ | b_2_ = (b_1_ + b_3_), & θ_12_ = θ_23_ = - b_2_ |
