# Supplementary material for: Life Course Socioeconomic Position: Associations with Cardiac Structure and Function at Age 60-64 Years in the 1946 British Birth Cohort
Source: PLoS One. 2016 Mar 31;11(3):e0152691. doi: 10.1371/journal.pone.0152691 (PMC4816291; doi:10.1371/journal.pone.0152691)
Supplement: S3 Table — (DOCX) [file pone.0152691.s003.docx]

**S3 Table:** Means (standard deviations) of cardiac measures by social class trajectory over the life course.

| **Life course  social class ^a^** | | | **N (%)** | **LV Structure** | | **Systolic function** | | **Diastolic function** | | |
| --- | --- | --- | --- | --- | --- | --- | --- | --- | --- | --- |
| **Child- hood** ^b^ | **Early Adult** ^c^ | **Middle Age** ^d^ |  | LV Mass Index (g/m ^2.7^) | RWT | EF | mFS (%) | LA volume index  (ml/m^2.7^) | E/A ratio | E/e’ ratio |
| **Men** | | | | | | | | | | |
| 0 | 0 | 0 | 275 (38.6) | 43.9 (13.2) | 0.43 (0.09) | 67.9 (10.2) | 16.7 (3.0) | 9.3 (3.0) | 1.03 (0.26) | 7.11 (1.84) |
| 0 | 0 | 1 | 15 (2.1) | 48.0 (14.4) | 0.43 (0.12) | 69.8 (6.8) | 17.1 (2.8) | 9.4 (4.1) | 1.18 (0.35) | 7.84 (1.61) |
| 0 | 1 | 0 | 35 (4.9) | 45.1 (12.8) | 0.39 (0.07) | 66.7 (9.0) | 17.2 (2.7) | 9.2 (2.7) | 0.97 (0.27) | 7.74 (2.84) |
| 0 | 1 | 1 | 26 (3.7) | 44.0 (11.2) | 0.40 (0.07) | 67.7 (10.7) | 17.6 (3.5) | 9.7 (4.2) | 0.94 (0.25) | 7.84 (2.15) |
| 1 | 0 | 0 | 142 (19.9) | 46.9 (13.7) | 0.42 (0.09) | 66.7 (10.7) | 16.4 (2.9) | 9.9 (4.0) | 0.99 (0.30) | 7.79 (1.97) |
| 1 | 0 | 1 | 15 (2.1) | 45.9 (15.4) | 0.45 (0.15) | 66.7 (9.8) | 15.2 (4.5) | 10.2 (3.4) | 0.93 (0.17) | 6.68 (1.45) |
| 1 | 1 | 0 | 73 (10.3) | 49.1 (15.0) | 0.43 (0.09) | 62.2 (14.3) | 16.6 (3.5) | 10.4 (3.4) | 0.97 (0.24) | 7.53 (2.04) |
| 1 | 1 | 1 | 131 (18.4) | 49.7 (15.7) | 0.41 (0.08) | 67.2 (11.4) | 16.7 (3.4) | 10.3 (3.5) | 0.98 (0.28) | 7.94 (2.24) |
|  | **Total** |  | **712** | **46.3 (14.1)** | **0.42 (0.09)** | **67.3 (10.4)** | **16.7 (3.2)** | **9.7 (3.4)** | **1.00 (0.27)** | **7.50 (2.05)** |
| **Women** | | | | | | | | | | |
| 0 | 0 | 0 | 307 (41.3) | 41.0 (11.8) | 0.41 (0.08) | 69.8 (9.3) | 17.7 (3.2) | 9.6 (3.5) | 1.01 (0.27) | 7.99 (1.90) |
| 0 | 0 | 1 | 30 (4.0) | 42.2 (15.2) | 0.44 (0.09) | 72.1 (9.3) | 17.3 (3.3) | 9.7 (3.6) | 1.09 (0.38) | 8.56 (2.20) |
| 0 | 1 | 0 | 28 (3.8) | 39.0 (14.8) | 0.40 (0.10) | 68.3 (8.2) | 17.4 (3.2) | 9.1 (3.1) | 1.19 (0.56) | 8.40 (1.92) |
| 0 | 1 | 1 | 8 (1.1) | 50.7 (17.1) | 0.47 (0.10) | 70.3 (10.2) | 16.4 (3.8) | 9.0 (3.5) | 0.99 (0.17) | 8.79 (2.38) |
| 1 | 0 | 0 | 225 (30.2) | 43.1 (11.8) | 0.42 (0.09) | 70.0 (10.3) | 17.3 (3.6) | 9.6 (3.5) | 0.97 (0.23) | 8.45 (2.23) |
| 1 | 0 | 1 | 42 (5.7) | 45.6 (10.8) | 0.43 (0.10) | 69.8 (10.6) | 17.0 (3.2) | 10.5 (4.5) | 0.90 (0.24) | 8.19 (1.76) |
| 1 | 1 | 0 | 44 (5.9) | 41.7 (12.7) | 0.39 (0.07) | 70.4 (9.1) | 18.1 (2.9) | 9.3 (3.5) | 0.99 (0.25) | 8.38 (2.19) |
| 1 | 1 | 1 | 60 (8.1) | 46.4 (14.7) | 0.39 (0.08) | 68.1 (12.2) | 17.6 (3.7) | 10.3 (4.1) | 0.91 (0.22) | 8.71 (2.48) |
|  | **Total** |  | **744** | **42.5 (12.5)** | **0.41 (0.09)** | **69.8 (9.9)** | **17.5 (3.4)** | **9.7 (3.6)** | **0.99 (0.28)** | **8.26 (2.08)** |

**^a^** Social class: 1 = manual; 0 = non-manual; ^b^ childhood = father’s social class at age 4 (or 11 or 15); ^c^ early adulthood = own social class at age 26 (or 36); ^d^ middle age = own social class at age 53 (or 43).
