# Supplementary material for: Life Course Socioeconomic Position: Associations with Cardiac Structure and Function at Age 60-64 Years in the 1946 British Birth Cohort
Source: PLoS One. 2016 Mar 31;11(3):e0152691. doi: 10.1371/journal.pone.0152691 (PMC4816291; doi:10.1371/journal.pone.0152691)
Supplement: S4 Table — (DOCX) [file pone.0152691.s004.docx]

**S4 Table:** Sex- and age-adjusted differences (95% CI) in cardiac markers by socioeconomic position at three points during the life course, after multiple imputation.

|  | LV Structure | | Systolic Function | | Diastolic Function | | |
| --- | --- | --- | --- | --- | --- | --- | --- |
|  | LV Mass Index (g/m ^2.7^) | RWT | EF | mFS (%) | LA volume index  (ml/m^2.7^) | E/A ratio | E/e’ ratio |
|  | (N = 1,477) | (N = 1,477) | (N = 1,491) | (N = 1,473) | (N = 1,406) | (N =1,575) | (N = 1,489) |
| **Childhood** | | | | | | | |
| I and II | – | – | – | – | – | – | – |
| IIInm | 0.29  (-1.63, 2.22) | 0.00  (-0.02, 0.01) | -0.34  (-1.78, 1.10) | -0.04  (-0.53, 0.44) | 0.75  (-1.45, 2.95) | 0.00  (-0.05, 0.04) | 0.09  (-0.20, 0.38) |
| IIIm | 3.89  (2.07, 5.71) | 0.00  (-0.01, 0.01) | -0.82  (-2.31, 0.68) | -0.26  (-0.76, 0.24) | 1.70  (-0.34, 3.73) | -0.06  (-0.10, -0.03) | 0.51  (0.24, 0.79) |
| IV and V | 4.12  (2.24, 6.00) | 0.00  (-0.01, 0.01) | -0.38  (-1.94, 1.17) | -0.16  (-0.67, 0.35) | 2.25  (0.11, 4.40) | -0.05  (-0.09, -0.01) | 0.43  (0.12, 0.74) |
| *Trend p* | *<0.0001* | *0.89* | *0.51* | *0.41* | *0.03* | *0.001* | *0.001* |
| **Early Adulthood** | | | | | | | |
| I and II | – | – | – | – | – | – | – |
| IIInm | 1.61  (-0.05, 3.26) | 0.00  (-0.01, 0.01) | 0.14  (-1.19, 1.47) | -0.12  (-0.55, 0.31) | 0.65  (-1.26, 2.56) | -0.02  (-0.05, 0.02) | 0.32  (0.04, 0.59) |
| IIIm | 3.64  (1.78, 5.49) | -0.02  (-0.03, 0.00) | 0.18  (-1.44, 1.80) | 0.42  (-0.10, 0.94) | 1.55  (-0.67, 3.76) | -0.02  (-0.06, 0.02) | 0.53  (0.22, 0.85) |
| IV and V | 3.73  (1.48, 5.97) | -0.01  (-0.03, 0.00) | -0.93  (-2.89, 1.02) | -0.18  (-0.79, 0.43) | 2.58  (0.06, 5.10) | -0.02  (-0.06, 0.02) | 0.36  (-0.01, 0.73) |
| *Trend p* | *<0.0001* | *0.04* | *0.50* | *0.80* | *0.03* | *0.25* | *0.005* |
| **Middle Age** | | | | | | | |
| I and II | – | – | – | – | – | – | – |
| IIInm | 1.51  (-0.23, 3.25) | 0.00  (-0.01, 0.02) | -0.46  (-1.82, 0.90) | -0.23  (-0.69, 0.22) | -0.57  (-2.59, 1.44) | -0.02  (-0.06, 0.02) | 0.16  (-0.11, 0.43) |
| IIIm | 4.04  (2.08, 5.99) | -0.01  (-0.02, 0.00) | -0.72  (-2.33, 0.88) | 0.14  (-0.40, 0.67) | 1.35  (-1.22, 3.92) | -0.04  (-0.08, 0.00) | 0.42  (0.08, 0.76) |
| IV and V | 4.69  (2.39, 6.99) | 0.01  (-0.01, 0.02) | -0.33  (-2.29, 1.63) | -0.28  (-0.87, 0.31) | 0.73  (1.73, 3.18) | -0.06  (-0.11, -0.01) | 0.43  (0.05, 0.82) |
| *Trend p* | *<0.0001* | *0.89* | *0.52* | *0.60* | *0.33* | *0.01* | *0.002* |

As well as the measures in the analysis models, the imputation model also included the following: birth weight; BMI at ages 4, 15, and 36 years; blood pressure at ages 36, 43 and 53 years; pack-years of cigarettes smoked from ages 20, 25, 31, 36, 43 and 53; triglycerides, glucose, insulin, and waist-to-hip ratio at age 60‑64; and response at the 2006‑2010 data collection. Multiple imputation models were restricted to participants who had a measured value of the outcome in question.
